# Variation in the Chemical Composition of Endemic Specimens of *Hedychium coronarium* J. Koenig from the Amazon and In Silico Investigation of the ADME/Tox Properties of the Major Compounds

**DOI:** 10.3390/plants12142626

**Published:** 2023-07-12

**Authors:** Jorddy Neves Cruz, Mozaniel Santana de Oliveira, Marcia Cascaes, Suraj N. Mali, Srushti Tambe, Cleydson Breno Rodrigues dos Santos, Maria das Graças Bichara Zoghbi, Eloisa Helena de Aguiar Andrade

**Affiliations:** 1 Adolpho Ducke Laboratory, Botany Coordination, Museu Paraense Emílio Goeldi, Belém 66075-110, Pará, Brazilcascaesmm@gmail.com (M.C.); zoghbi@museu-goeldi.br (M.d.G.B.Z.); eloisa@museu-goeldi.br (E.H.d.A.A.); 2 Department of Pharmaceutical Sciences and Technology, Institute of Chemical Technology, Main Campus at Mumbai, Deemed University, Nathalal Parekh Marg, Mumbai 400019, Maharashtra, Indiasrushtitambe7@gmail.com (S.T.); 3 Laboratory of Modeling and Computational Chemistry, Department of Biological and Health Sciences, Federal University of Amapá, Macapá 68902-280, Amapá, Brazil; breno@unifap.br; 4 Faculty of Chemistry, Federal University of Pará, Rua Augusto Corrêa, Belém 66075-750, Pará, Brazil

**Keywords:** Zingiberaceae, *Hedychium coronarium*, essential oil, biological activities

## Abstract

Four species of the genus *Hedychium* can be found in Brazil. *Hedychium coronarium* is a species endemic to India and Brazil. In this paper, we collected six specimens of *H. coronarium* for evaluation of their volatile chemical profiles. For this, the essential oils of these specimens were extracted using hydrodistillation from plant samples collected in the state of Pará, Brazil, belonging to the Amazon region in the north of the country. Substance compounds were identified with GC/MS. The most abundant constituent identified in the rhizome and root oils was 1,8-cineole (rhizome: 35.0–66.1%; root: 19.6–20.8%). Leaf blade oil was rich in β-pinene (31.6%) and (E)-caryophyllene (31.6%). The results from this paper allow for greater knowledge about the volatile chemical profile of *H. coronarium* specimens, in addition to disseminating knowledge about the volatile compounds present in plant species in the Amazon region.

## 1. Introduction

Zingiberaceae has more than 50 genera and about 1400 plants [1]. The species that occur in Brazil are distributed in eight genera (*Alpinia* L., *Amomum Roxb.*, *Curcuma* L., *Etlingera* Giseke, *Hedychium coronarium* J. Koenig, *Kaempferia* L., *Renealmia* L.f e *Zingiber* Boehm) and, among these, the genus Hedychium stands out for having the second largest number of representatives in Brazil (four species) [2].

The species *Hedychium coronarium* J. Koenig is endemic to India and China. In Brazil it is considered invasive and is popularly known as “lily of the marsh”, “butterfly lily”, “white-lily garland”, “narcissus”, “napoleon”, “Olympia” or “white ginger”, in addition to having several uses in folk medicine [3,4].

*Hedychium* species are cultivated as medicinal plants, ornamental plants, spices, and condiments [5]. The population of Malaysia uses this species for the treatment of gastric disorders, such as indigestion [6], and the medicinal drink is produced in the form of tea by infusion or decoction [7]. In Thailand, they use the tea from this plant, produced by infusion, for the treatment of osteoarthritis, caused by the wear and tear of cartilage in the joints, in addition to using the tea produced with the stem to treat tonsillitis. The rhizome of the plant has several medicinal properties, being used in Thailand to combat the excessive amount of gases produced after digestion [8]. In Vietnam it is used as a healing treatment and an antiseptic, fighting bacteria in the case of wounds. In Brazil it is consumed as a diuretic or to treat hypertension [9]. EOs extracted from the rhizome of *H. coronarium* have shown anthelmintic activities [10,11] and antimicrobial activities [12], in addition to having a phytotoxic effect [13]. For the leaves and also the rhizomes, fibrinogenolytic, coagulant [14], larvicidal [15], and antioxidant [16,17,18] activities have been found.

In Hawaii, the flowers are eaten as vegetables and used as garlands, and in Japan, they are used for perfume production.

The chemical composition of EOs from *H. coronarium* depend on where the species is collected. Previous studies showed that the EO from the leaves of a specimen collected in Taiwan presented as major constituents β-pinene (33.9%), α-pinene (14.7%), and 1,8-cineole (13.3%), while in the rhizomes, we found as the majority 1,8-cineole (37.3%), β-pinene (23.0%), and α-terpineol (10.4%) [19]. The rhizome EO collected in India was characterized by eucalyptol (37.6%), p-cymene (11.6%), and p-menth-1-en-8-ol (9.4%). On the other hand, the rhizome EO of a specimen collected in Brazil showed 1,8-cineole (33.5%), β-pinene (17.0%), and α-terpineol (7.7%) as the main constituents [20,21]. A specimen collected in Ecuador had the EO characterized by 1,8-cineole (33.7%), β-pinene (30.0%), and α-pinene (10.0%) [16].

Considering that EOs can lead to the discovery of new chemotypes of a species and that these variations are influenced by geographic and environmental factors [22,23,24,25], the present work aims to evaluate the chemical composition of the EOs for six specimens of *H. coronarium* collected in different municipalities of the state of Pará, Brazil. We aim to provide valuable scientific data to enhance the understanding of volatile compound profiles in the Amazon region. Furthermore, our data significantly support the comprehension of new specimens of *H. coronarium*. Through a comprehensive and rigorous approach, we innovatively investigated the diversity and composition of volatile compounds present in this unique region.

## 2. Results and Discussion

### 2.1. Chemical Composition

Table 1 and Table 2 show the 65 chemical compounds found in the EOs of the six specimens. The EO of specimen A, collected on the border between the states of Pará–Maranhão, Brazil, was characterized by 1,8-cineole (66.1%) and β-pinene (21.9%) in the rhizomes; on the other hand, the leaves from this species showed a majority of β-pinene (48.9%) and 1,8-cineole (66.1%). A majority of 1,8-cineole (46.2%) and β-pinene (31.1%) characterized, respectively, the EOs of the rhizomes and leaves from specimen B, collected in the municipality of Tracuateua, Pará, Brazil. In the EO of sample C, collected in the municipality of Igarapé-Miri, Pará, Brazil, 1,8-cineole (37.4%) in the rhizomes and β-pinene (34.8%) in the leaves were found in the majority. In specimen D, collected in Santarém Novo, Pará, Brazil, the EO was characterized by 1,8-cineole (35%) in the rhizomes and β-pinene (31.6%) in the leaves. In Belém, Pará, Brazil, two samples were collected (E and F), the EO of sample E presented the majority as β-pinene in the rhizomes (30.5%) and in the leaves (41%), while sample F presented 1,8-cineole (33.5%) in rhizomes and α-pinene in the leaves (32.9%).

The results found in this study showed differences between them that may be associated with the climate, collection period, collection sites, and ecosystems [26,27]. Furthermore, our results differed from the findings for the EO extracted from the rhizomes of a species collected in India that was characterized by the majorities: β-pinene (11.07–42.74%), eucalyptol (11.48–40.59%), linalool (1.56–45.11%), coronarin E (1.01–39.57%), α-pinene (3.80–16.60%), p-cymene (1.05–8.89%), γ-terpinene (1.73–5.82%), and 10-epi-γ-eudesmol (1.11–4.86%) [12]. The contents of the monoterpene compounds, α-pinene and β-pinene, in this work were lower than those in our study.

In another study by Ray et al., 2017 [28], the EO of the rhizomes from a specimen of *H. coronarium* collected in India showed the following major compounds: eucalyptol (37.62%), p-cymene (11.68%), and p-menth-1-en-8-ol (9.44%). While in the research carried out by Prakash et al., 2012 [29], the EO of the rhizomes presented the following main components: linalool (29.3%) limonene (20.3%), trans-m-mentha 2,8-diene (12.9%), γ-terpinene (8.9%), camphene (3.7%), α- pinene (3.5%), 10-epi-γ-eudesmol (3.7%), and ar-curcumene (2.7%). The major compounds α-pinene (20.0%), linalool (15.8%), 1,8-cineole (10.7%), α-pinene (10.1%), and α-terpineol (8.6%), characterized the EO of the leaves from *H. coronarium* collected in Vietnam [30].

The monoterpene compound 1,8-cineole is the main constituent present in EOs of *H. coronarium* [31], and studies report that this compound has activity against human colon cancer cells HCT116 [32], antimicrobial action against fungi [33] of the type *Trichoderma* sp. and *Candida albicans*, *Bacillus subtilis* and *Pseudomonas aeruginosa* [34,35], anti-inflammatory action against acute pancreatitis [36], as well as antioxidant, sedative, antiviral, anesthetic, and analgesic properties [37].

β-pinene is one of the major compounds that showed significant significance in the results. This hydrocarbon monoterpene is found in the EOs of many coniferous plants, such as pine (*Araucaria angustifolia*) [38], and some research involving EOs has shown that this compound has antioxidant properties, biological activities against bacteria (*Acetobacter calcoacetica*, *Bacillus subtilis*, *Clostridium sporogenes*, *Clostridium perfringens*, *Escherichia coli*, *Salmonella typhi*, *Staphylococcus aureus,* and *Yersinia enterocolitica*) and fungi (*Candida albicans*, *Aspergillus niger*, *Aspergillus flavus* and *Penicillium notatum*) [39]. In turn, α-pinene is described in the literature as having a modulating action of antibiotic resistance against the multidrug-resistant bacterium *Campylobacter jejuni* that causes gastroenteritis [40,41], and studies report that α-pinene has a greater antimalarial property than β-pinene [41].

The sesquiterpenes (E)-caryophyllene and caryophyllene oxide showed significant levels in the EOs of the leaves from sample D, the first compound with a content of 20% and the second with 10.4%. (E)-caryophyllene is one of the main active compounds present in the EOs of food plants and spices, such as basil (*Ocimum* spp.), cinnamon (*Cinnamomum* spp.), black pepper (*Piper nigrum*), cloves (*Syzygium aromaticum*), cannabis (*Cannabis sativa*), lavender (*Lavandula angustifolia*), oregano (*Origanum vulgare* L.), and rosemary (*Rosmarinus officinalis*) [42]. This compound has anesthetic potential [43], is cytotoxic against MCF-7, DLD-1, and L-929 cell lines [44], is anti-inflammatory [45], and anticonvulsant [46].

Caryophyllene oxide is a low water solubility compound with a strong wood odor and is even used as a food additive [42]. The said sesquiterpene has cytotoxic potential against HepG2, AGS, HeLa, SNU-1, and SNU-16 cancer cells [47], as well as anti-inflammatory potential [48], antioxidant [49], antiviral [50], and analgesic properties [42,51].

Regarding the specific question on whether the chemotypes of the leaves and rhizomes were the same for the same sample, our research indicates that there are variations in the chemical composition of both plant parts within a single specimen. The differences observed between the leaves and rhizomes highlight the importance of considering different plant organs when studying volatile compounds in *H. coronarium*. These findings suggest that the biosynthesis and accumulation of volatile compounds may be organ specific, indicating potential variations in their ecological roles and chemical profiles.

**Table 1:** Chemical composition of the oils derived from the rhizomes.

RI_L_	RI_C_	Constituents	A1	B1	C1	D1	E1	F1
924	930	α-thujene	0	0.6	0.7	0.6	0.7	0
932	938	α-pinene	4.5	9.1	11.5	9.6	12.5	19.6
946	952	camphene	0	0.7	0.8	0.7	0.9	0
969	975	sabinene	0.6	1.3	1.3	2.3	2.6	0
974	979	β-pinene	21.9	23	28.9	25.9	30.5	7.6
988	990	myrcene	0.3	1.3	1.5	1.4	2.3	10.7
1002	1003	α-phellandrene	0	0	2.3	3.4	0	10.3
1003	1004	p-mentha-1(7),8-diene	0	0	0.4	0	0	0
1014	1017	α-terpinene	0	0.4	0.6	0.4	0.7	1.5
1020	1027	p-cymene	0	0.8	0.5	1.3	0.5	0
1024	1029	Limonene	0	3	3.3	3.5	4.4	0
1026	1032	1,8-cineole	66.1	46.2	37.4	35	26	33.5
1036	1036	phenyl acetaldehyde	0	0.1	0	0.1	0.2	0
1044	1048	(E)-β-ocimene	0	0	0	0	0	0.2
1054	1060	γ-terpinene	0.5	0.9	1.1	1.1	1.6	3.4
1086	1089	terpinolene	0	0.3	0.4	0.4	0	1.2
1095	1097	linalool	1.1	0.1	0.9	0.2	0.3	0.5
1098	1099	(E)-sabinene hydrate	0	0	0	0.7	0.1	0
1114	1117	endo-fenchol	0	0	0	0	0	0.1
1128	1126	α-campholenal	0	0	0	0.1	0	0
1132	1131	allocymene	0	0	0	0	0	0
1137	1137	(E)-limonene oxide	0	0.1	0	0.3	0	0
1135	1140	trans-pinocarveol	0	0	0	0.1	0	0.2
1141	1142	Camphor	0	0.1	0.9	0.2	0.3	0.1
1165	1166	borneol	0.2	0.6	0.8	1.2	0.6	1.8
1174	1178	terpinen-4-ol	1.5	1.8	2.1	1.8	1.2	2.7
1179	1179	p-cymen-8-ol	0	0	0	0.1	0	0
1186	1190	α-terpineol	3.3	3.1	4.1	5.2	2.8	4.2
1194	1195	myrtenol	0	0	0	0	0	0.2
1284	1289	bornyl acetate	0	0	0	0	0.1	0.2
1335	1340	δ-elemene	0	0	0	0.7	0.1	0
1346	1351	α-terpinyl acetate	0	0	0	0.1	0	0.3
1417	1417	(E)-caryophyllene	0	0.1	0	0.3	0.3	0.5
1452	1455	α-humulene	0	0	0	0	0	0.1
1505	1506	β-bisabolene	0	0	0	0.4	0.1	0
1582	1580	caryophyllene oxide	0	0	0	0.1	0.1	0.3
1640	1644	epi-α-muurolol	0	0	0	0.1	0.1	0
1759	1763	benzyl benzoate	0	0	0.3	0	0	0
		Hydrocarbon monoterpenes	27.8	41.4	53.3	50.6	56.7	54.5
		Oxygenated monoterpenes	72.2	52.0	46.2	45.0	31.4	43.8
		Hydrocarbon sesquiterpenes	0	0.1	0	1.4	0.5	0.6
		Oxygenated sesquiterpenes	0	0	0	0.2	0.2	0.3
		Other Class		0.1	0.3	0.2	0.2	0
		Total	100	93.6	99.8	97.4	89.9	99.2

RI_L_: literature retention index [52]; RI_C_: retention index (on DB-5MS column).

**Table 2:** Chemical composition of the oils derived from the leaves.

RI_L_	RI_C_	Constituents	A2	B2	C2	D2	E2	F2
924	930	α-thujene	0.1	0.4	0.4	0.3	0.5	0
932	938	α-pinene	11.6	16.3	15.9	14.7	20.7	32.9
946	952	camphene	0.1	0.3	0.3	0.2	0.2	0
969	975	sabinene	0.2	1.6	3.1	2.7	5.7	0
974	979	β-pinene	48.9	31.1	34.8	31.6	41	9.9
988	990	myrcene	0.2	0.5	0.7	0.5	1.1	5.2
1001	1001	δ-2-carene	0	0	0	0	0	0.1
1002	1003	α-phellandrene	0	0.1	0.2	0	0	0
1014	1017	α-terpinene	0.2	0.5	0.3	0.4	0.2	0.4
1020	1027	p-cymene	0.1	0.3	0.2	0.2	0.1	0
1024	1029	Limonene	0	1.6	1.9	1.5	1.9	4.5
1026	1032	1,8-cineole	16.9	2.2	5.5	1.9	2.7	8.9
1036	1036	phenyl acetaldehyde	0	0.2	0	0.3	0.6	0
1044	1048	(E)-β-ocimene	0	0	0.2	0	0	1.1
1054	1060	γ-terpinene	0.5	0.9	0.7	0.7	0.4	1.2
1086	1089	terpinolene	0	0.3	0.2	0.2	0.1	0.5
1095	1097	linalool	1.1	0.1	0	0.1	0	0
1098	1099	(E)-sabinene hydrate	0	0.1	0	0	0.1	0
1128	1126	α-campholenal	0	0	0	0.2	0.1	0
1132	1131	allocymene	0	0	0	0	0	0.8
1137	1137	(E)-limonene oxide	0	0.7	0	0.2	0	0
1135	1140	trans-pinocarveol	0.1	0.1	0.4	0	0	0.4
1141	1142	camphor	1.1	0.1	0	0.1	0	0
1140	1145	trans-verbenol	0	0.1	0.4	0	0	0
1160	1163	pinocarvone	0.3	0.1	0.4	0	0	0.7
1165	1166	borneol	0.3	0.6	0	0.1	0.1	0
1174	1178	terpinen-4-ol	0.7	1.5	0.9	0.1	0.4	1
1179	1179	p-cymen-8-ol	0	0.1	0	0.1	0	0
1186	1190	α-terpineol	2	2.7	1.6	0.7	0.4	0.5
1194	1195	myrtenol	0	0	0	0	0	0.6
1204	1205	verbenone	0	0.1	0	0	0	0
1284	1289	bornyl acetate	0	0.1	0	0	0.1	0.1
1285	1292	safrole	0	0	0	0	0	0.1
1298	1298	(E)-pinocarvyl acetate	0	0.2	0	0	0	0
1335	1340	δ-elemene	0	0.1	0	0	0.1	0.3
1346	1351	α-terpinyl acetate	0.1	0	0	0.2	0.1	0
1356	1361	eugenol	0	0	0	0	0	0.1
1389	1392	β-elemene	0	0.6	0	0	0	0.2
1417	1417	(E)-caryophyllene	3.1	15.1	13.2	20	14.1	10.5
1428	1432	(E)-α-ionone	0	0	0	0	0	0.1
1434	1437	γ-elemene	0	0	0	0	0	0.1
1442	1448	guaia-6,9-diene	0	0	0	0	0	0.1
1452	1455	α-humulene	0.3	1	0.9	1.4	0	1.5
1454	1457	(E)-β-farnesene	0	0.3	0	0	0.3	0
1480	1486	germacrene D	0	0	0	0	0	0.1
1487	1491	(E)-β-ionone	0	0	0	0	0	0.1
1505	1506	β-bisabolene	0	0.1	0	0	0.1	0
1513	1515	γ-cadinene	0	0	0	0	0	0.1
1520	1522	7-epi-α-selinene	0	0	0	0.2	0	0
1522	1527	δ-cadinene	0	0	0	0	0	0.1
1561	1563	(E)-nerolidol	0	0.3	0.5	0.9	0.3	0.1
1577	1577	spathulenol	0	1.2	1.1	1.3	0	0
1582	1580	caryophyllene oxide	5.7	10	5.3	10.4	2.8	4.9
1608	1605	humulene epoxide II	0	0.9	0.5	1	0.2	0.5
1620	1624	dillapiole	0	1	0.3	1.1	0.4	0.1
1627	1632	1-epi-cubenol	0	1.2	0	0	0	0
1638	1640	epi-α-cadinol	0	1.6	1	1.1	0	0
1639	1641	cariophylla-(12),8(13)-dien-5α-ol	0	0	0	0	0	0.3
1639	1642	cariophylla-(12),8(13)-dien-5β-ol	0	0	0	0	0	0.9
1640	1644	epi-α-muurolol	0	0.6	2	2.1	0.2	0
1644	1645	α-muurolol	0	1.4	0	0.2	0	0
1652	1654	α-cadinol	0	0	0.5	0	0	0.1
1759	1763	benzyl benzoate	0.3	0	0.2	0	0	0
		Hydrocarbon monoterpenes	61.9	53.9	58.9	53.0	71.9	55.8
		Oxygenated monoterpenes	22.6	8.8	9.2	3.7	4.0	12.4
		Hydrocarbon sesquiterpenes	3.4	17.2	14.1	21.6	14.6	13.0
		Oxygenated sesquiterpenes	5.7	17.2	10.9	17.0	3.5	6.8
		Other Class	0.3	1.2	0.5	1.4	1.0	0.3
		Total	93.9	98.3	93.6	96.7	95.0	88.3

RI_L_: literature retention index [52]; RI_C_: retention index (on DB-5MS column).

### 2.2. Multivariate Analysis

**Figure 1:**
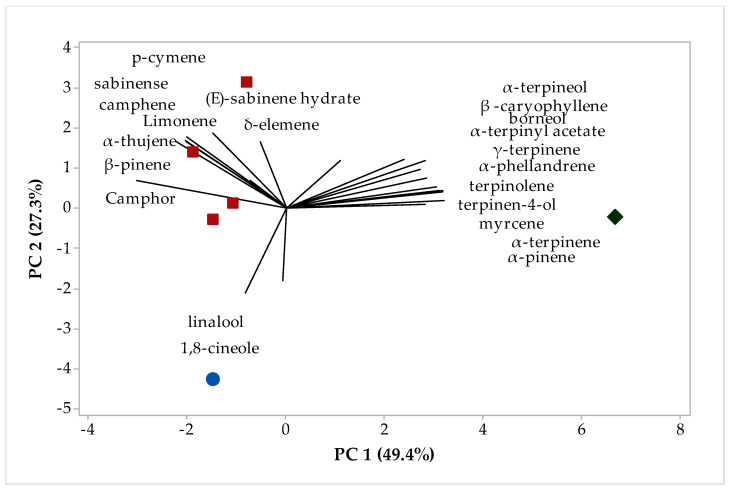
Biplot (PCA-rhizomes) results from the analysis of compounds identified in the *H. coronarium* EO.

**Figure 2:**
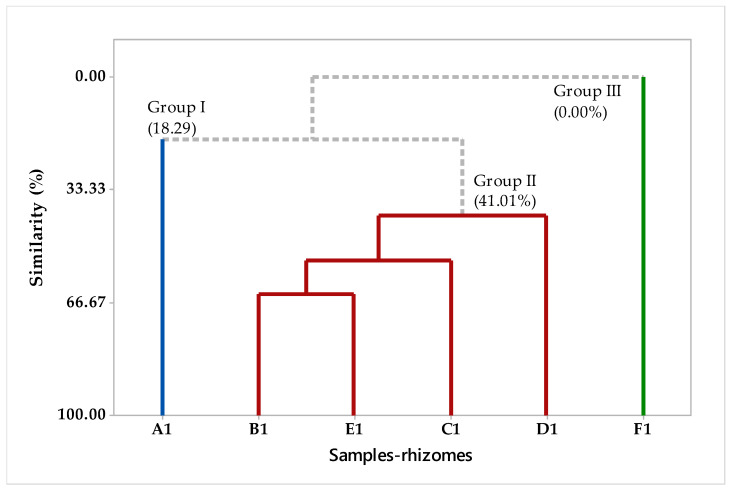
The dendrogram (HCA-rhizomes) represents a similar relationship to the compounds identified in the *H. coronarium* EO.

The chemical compounds were identified in the different fractions of EOs of *H. coronarium*. The multivariate analysis PCA (principal component analysis) is shown in Figure 1 and the HCA (hierarchical cluster analysis) is shown in Figure 2. In Figure 1, we can see that PC1 explains 49.4%, while PC2 explains 27.3% of the variations, and the two components add up to 76.7% of the variance. When analyzing the HCA, considering the Euclidean distances and complete bonds (Figure 2), we have the formation of three distinct groups formed by the fractions, with group I formed only by sample A1, while group II is formed by samples B1, E1, C1, and D1, with a similarity of 41.01% (Figure 2), while group III did not show a significant level of similarity with any sample rhizome EO.

In addition, Figure 1 shows the compounds that each characteristic group formed in the multivariate analysis, for example, group I, which comprises the largest number of grouped samples, was formed by the compounds 1,8-cineole and linalool. On the other hand, in group II, the compounds that contributed positively to the similarity between the different fractions were p-cymene, sabinene limonene, camphene, (E)-sabinene hydrate, d-elemene, a-thujene, b-pinene, and camphor, and in group III α-terpineol, (E)-caryophyllene, borneol α-terpinyl acetate γ-terpinene, α-phellandrene, terpinolene, terpinen-4-ol, myrcene, α-terpinene, and α-pinene (Figure 1).

**Figure 3:**
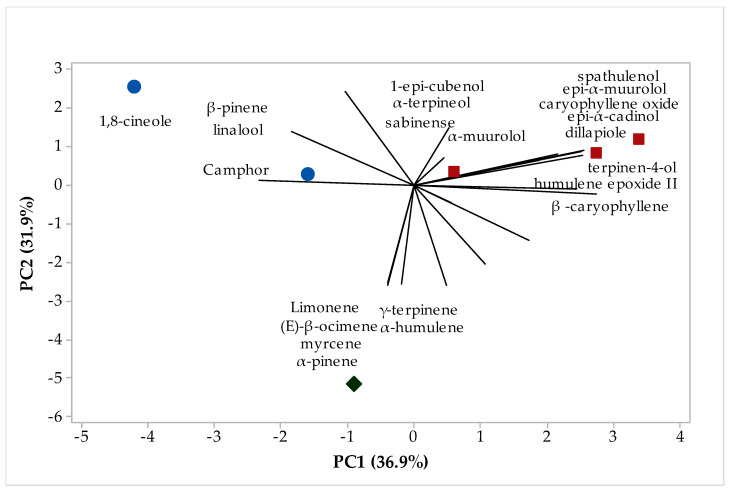
Biplot (PCA-leaves) results from the analysis of compounds identified in the *H. coronarium* EO.

**Figure 4:**
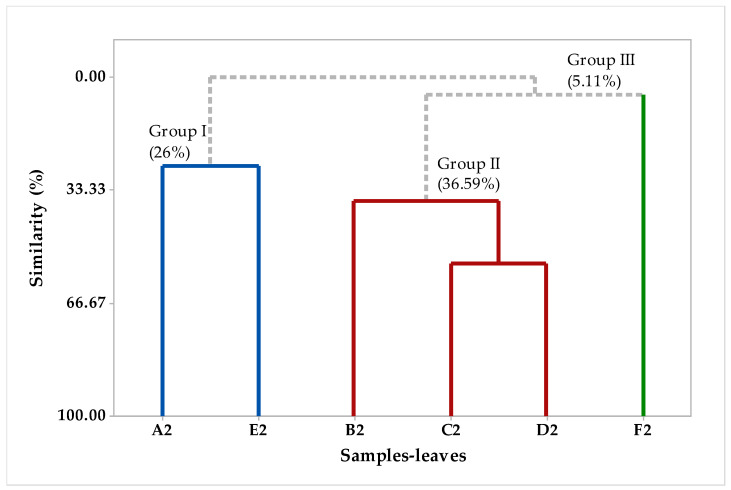
The dendrogram (HCA-leaves) represents a similar relationship to the compounds identified in the *H. coronarium* EO.

A multivariate analysis was applied to analyze the similarity in the chemical composition between the different fractions of EOs isolated from the *H. coronarium* leaves. Figure 3 shows the principal component analysis (PCA), while Figure 4 shows the hierarchical cluster analysis (HCA), according to which we can observe with the results obtained in the PCA (Figure 3) that the first component explains 36.9%, while PC2 explains 31.9% of the variances, the sum of the two components explains 68.8% of the variations observed in Figure 4 of the HCA. We note that there was the formation of three groups, the first group was formed by the samples of oils A2 and E2, group II was formed by the samples B2, C2, and D2, while group III was formed only by the sample F2, with a similarity of 26% of samples A2 and B2, 36.59% between samples B2, C2, and D2, and 5.11% of sample III in relation to sample II, that is, a low similarity between them (Figure 4). In addition, in Figure 3, it is possible to observe which compounds were responsible for positively or negatively impacting the formation groups, for example in group I the highest number of compounds were 1,8-cineole, β-pinene, and camphor, in group II they were 1-epi-cubenol, α-terpineol, sabinene, α-muurolol, spathulenol, epi-a-muurolol, caryophyllene oxide, epi-α-cadinol, terpi-ne-4-ol, humulene epoxide II, and (E)-caryophyllene, while in group III they were limonene y-terpinene, (E)-β-cimene, α-humulene, myrcene, and α-pinene. Chemometric analysis has been shown to be an important tool for researchers of natural products, because through it they can analyze the differences between samples of EOs using matrix correlation, which shows the differences and similarities between samples of different plants or samples collected at different times, or different regions of ions [53].

**Figure 5:**
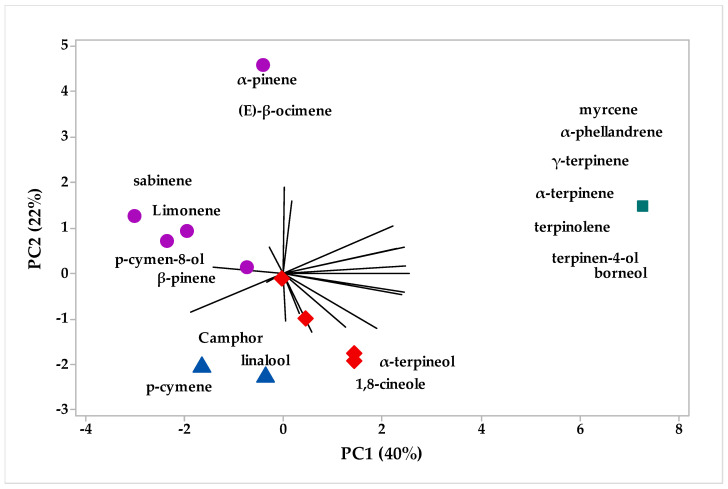
Biplot (PCA-compounds-leaves and Rhizome) results from the analysis of compounds identified in the *H. coronarium* EO.

**Figure 6:**
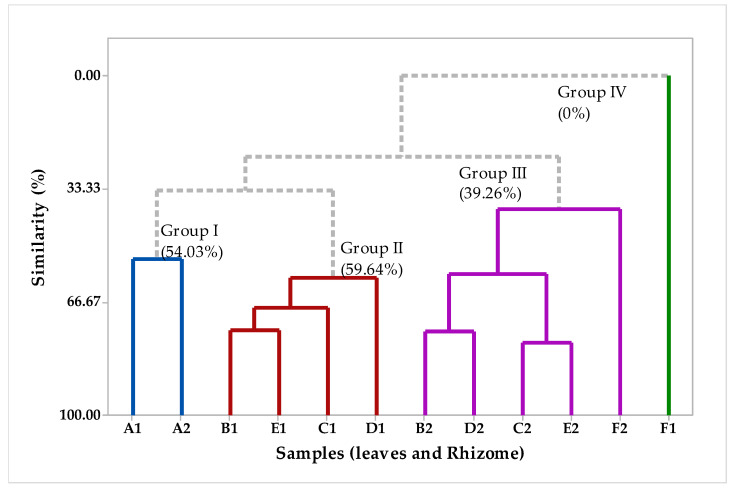
The dendrogram (HCA compounds-leaves and Rhizome) represents a similar relationship to the compounds identified in the *H. coronarium* EO.

Multivariate analysis was used to verify the potential similarity of the different fractions of EOs obtained from the vegetative organs, rhizomes, and leaves of *H. coronarium*. In addition, we can see in the graph that each component explains a value of the variance in the analyzed data, for example, the first component explains 40% and the second component explains 22% of the variance (Figure 5). In the HCA hierarchy analysis, Figure 6, we can analyze the formation of the different groups. In general, there was the formation of four groups, with different degrees of similarity. Group I, with a similarity of 54.03%, was formed by the samples A1 and A2, essential oils from the rhizome and leaves, respectively. Group II was formed by samples of the EO only isolated from the rhizome, namely B1, E1, C1, and D1, with a similarity of 59.64%. Group III, with a similarity of 39.26%, was formed only by a sample of essential oils isolated from the leaves, namely B2, D2, E2, and F2. The sample F1 group IV followed the same pattern already described in Figure 2, that is, it had no similarity with the other samples. These results demonstrate that plant organs can biosynthesize different substances in qualitative and quantitative terms.

**Figure 7:**
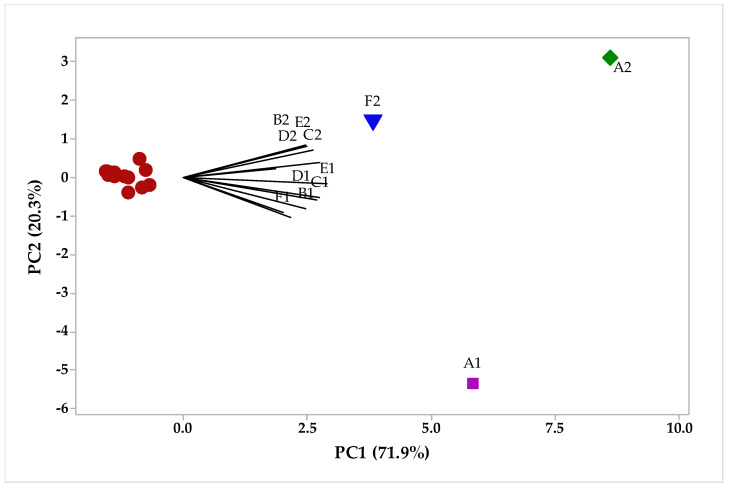
Biplot (PCA-leaves and Rhizome) results from the analysis of compounds identified in the *H. coronarium* EO.

**Figure 8:**
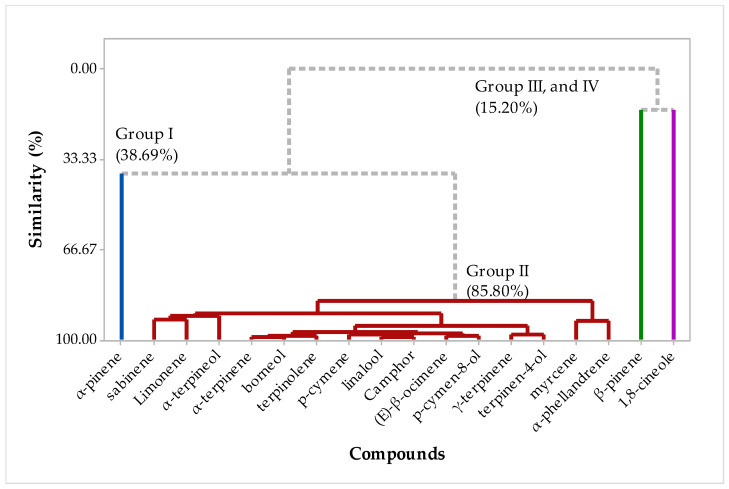
The dendrogram (HCA-leaves and Rhizome) represents a similar relationship to the compounds identified in the *H. coronarium* EO.

The multivariate PCA analyses were carried out in the factorial plane for the samples of essential oils from the leaves and rhizomes. In the PCA, we can observe that PC1 explains 71.9% of the variance and PC2 explains 20.3%. In Figure 7, it is possible to analyze that three oil samples are separated from F2, A1, and A2, corroborating the previous results of the chemometric analysis for the compounds, Figure 3 and Figure 5. In addition, a HCA hierarchy analysis (Figure 8) was carried out, the results of which corroborate those presented in all the previous graphs; for example, the compounds that had the highest weights for the formation of the groups, the group was formed only by the F2 sample, this may be related to the presence of α-pinene in a higher concentration. Group II was formed by the other samples (B1, C1, D1, E1, F1, A2, B2, C2, D2, and E2), and the relationship between them is in the presence of the compounds sabinene, β-pinene, myrcene, α-phellandrene, α -terpinene, p-cymene, Limonene, 1, 8-cineole, (E)-β-ocimene, γ-terpinene, terpinolene, linalool, Camphor, borneol, terpinen-4-ol, p-cymen-8-ol, and α-terpineol. Group III and IV are formed by separate samples A2 and A1, with the most representative compounds β-pinene and 1,8-cineole, respectively, being in agreement with the HCA analysis, as shown in Figure 4 and Figure 6.

### 2.3. In Silico ADMET Analysis

Due to the limited pharmacokinetics and metabolic performance of essential oils, they often fail to meet the requirements for antimicrobial/antibacterial drug testing [54,55,56]. Therefore, we conducted an analysis of the ADMET profile for the main constituents found in the tested essential oils. Our analysis retained the calculations of more than 50 ADMET parameters for the studied compounds, namely 1,8-Cineole, α-Pinene, β-pinene, and (E)-caryophyllene.

Table 3 provides an overview of the estimated ADMET properties for the selected compounds. Lipinski’s Rule of Five, introduced by Dr. Christopher Lipinski, is a guideline in drug design. It assesses a compound’s oral bioavailability based on its molecular weight, lipophilicity, hydrogen bond donors, and acceptors. These criteria help determine a compound’s drug-likeness and potential for successful oral administration. In accordance with important drug-likeness guidelines, a compound should not violate more than one Lipinski rule. Furthermore, its molecular weight should be below 500 g/mol, its topological surface area (TPSA) should be less than 140 Å^2^, the number of H-bond acceptors (nOHA) should not exceed five, the number of H-bond donors (nOHD) should be five or less, the water partition coefficient (WLOGP) should not exceed 5.88, and the number of rotatable bonds (nRB) should be ten or less [57,58]. As per Table 3, those compounds violating more than one parameter would be considered as a Lipinski violation. Based on our findings, all the compounds had a TPAS less than 30 Å^2^. Except for α-pinene, β-pinene, and (E)-caryophyllene, all the compounds exhibited high gastrointestinal absorption (GI), indicating their easy absorption through the gastrointestinal tract. Many of the compounds were found to be (theoretically) soluble in water (except terpenes and sesquiterpenes), which is an important criterion for their effectiveness as a drug. One of the major components of the EO, α-pinene is a colorless, water-insoluble, but oil- and ethanol-soluble organic liquid. β-pinene is also a colorless organic liquid, which is oil soluble but ethanol- and water-insoluble. Moreover, 1,8-cineole is insoluble in water, 3.50 × 10^3^ mg/L at 21 °C, and also miscible with ether, alcohol, chloroform, glacial acetic acid, and oils. As per the data available from the National Institutes of Health (NTP), 1992, it is insoluble at <1 mg/mL at 68 °F. However, data published by the Joint FAO/WHO Expert Committee on Food Additives (JECFA) states that it is insoluble in water and miscible in oils. However, there is different information about this on the PubChem website (https://pubchem.ncbi.nlm.nih.gov/compound/Eucalyptol#section=Solubility, accessed on 1 July 2023). (E)-caryophyllene is soluble in ether and ethanol, and insoluble in water.

**Figure 9:**
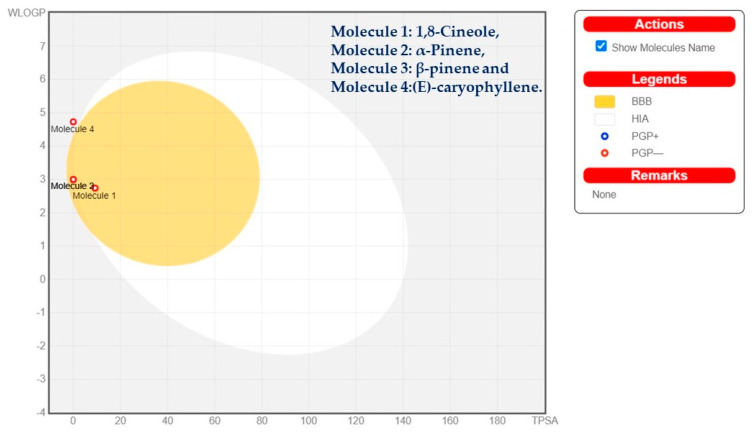
Boiled-egg graph of the selected phytoconstituents.

**Figure 10:**
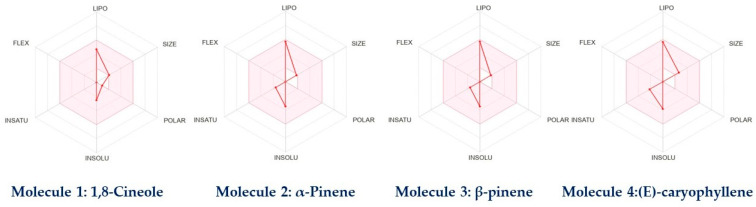
Bioavailability radar of the selected phytoconstituents (pink area shows the drug likeness properties of the selected compounds).

During the absorption process, first-pass metabolism via P-glycoprotein (P-gp) and cytochrome P450 enzymes in the small intestine and liver can negatively impact drug bioavailability. However, our results indicated no P-glycoprotein (P-gp) substrates among the compounds, suggesting good intestinal absorption, while some compounds mainly interacted with two isoenzymes of the cytochrome (CYP450) family, specifically CYP2C19 and CYP2C9, indicating their effectiveness with minimal toxicity. (E)-caryophyllene was predicted to be unable to cross the blood–brain barrier (BBB), as shown in the boiled-egg prediction. Compounds located in the yellow zone of the graph can permeate through the blood–brain barrier (BBB). The drug-like properties and gastrointestinal (GI) absorption of the chosen compounds from the essential oils were assessed using the boiled-egg prediction (Figure 9) and bioavailability radar graph (Figure 10). Compounds located in the yellow zone of the boiled-egg graph have the ability to cross the blood–brain barrier (BBB), while the pink area on the bioavailability radar graph indicates their drug-like characteristics.

Additionally, the toxicological properties of the compounds were assessed and presented in Table 3. None of the selected compounds exhibited organ or oral toxicity, except for (E)-caryophyllene. In summary, based on the results, it can be concluded that these compounds have the potential for further development as drug candidates. The LD_50_ values were also calculated to ensure the safety of the selected compounds, as shown in Table 3. The compounds with LD_50_ > 2000 mg/kg suggest their safety for biological administration and as potential drugs.

Our in silico results for the major compounds of different *H. coronarium* essential oils match with earlier reported data [59].

## 3. Materials and Methods

### 3.1. Material

Samples A–F of *H. coronarium* were collected in the state of Pará: Sample A (highway Pará–Maranhão Km 290), Sample B (municipality of Tracuateua), Sample C (municipality of Igarapé-Miri), Sample D (municipality of Santarém Novo), Samples E and F (Belém). Voucher specimens were deposited in the herbarium at the Museu Paraense Emílio Goeldi (Sample B: MG182,830, Sample E: MG182,843, and Sample F: MG177,796). The other samples were identified by comparison with authentic voucher plants.

### 3.2. Preparation of the Botanical Material

The A–F samples of *H. coronarium* leaves were dried in an oven with air circulation at 35 °C for five days and then ground in a knife mill (Tecnal, model TE-631/3, Piracicaba, São Paulo, Brazil).

### 3.3. Extraction of Volatile Compounds

The samples were subjected to hydrodistillation in modified Clevenger-type glass systems for 3 h, coupled with a refrigeration system to maintain the condensation water at around 12 °C, following protocols reported earlier by our research group [4,60].

### 3.4. Analysis of the Volatiles

The phytochemical profiles of the EOs were analyzed using chromatography/mass spectrometry (GC/MS) using a Shimadzu QP Plus 2010 GC-MS (Kyoto, Japan), following protocols reported earlier by our research group [4,60]. The retention index was calculated for all the volatile constituents using a homologous series of n-alkanes (C8-C40, Sigma-Aldrich, St. Louis, MO, USA), according to Van den Dool and Kratz [61], and the compounds were identified by comparing their mass spectrum and retention index with the data from the libraries [52].

### 3.5. ADMET Analyses

In modern drug-like hit identification, estimations of the pharmacokinetic properties have a crucial role in it [62,63]. Nowadays, many machine learning-based theoretical ADMET analyses tools are available online, which helps scientists to get more insights from these properties before actually going for higher pre-clinical studies. Although they have their own limitations, certainly the tools with good applicability domains have higher chances of accurate predictions. ‘SwissADME’ is one of the tools available online, which is useful for theoretical ADMET assessments [64,65]. One important mechanism that underpins drug–drug interactions is the induction or inhibition of CYP enzymes. Considering this fact, our in silico analyses for CYP1A2 inhibition, CYP2C19 inhibition, CYP2C9 inhibition, CYP2C9 substrate, CYP2D6 inhibition, CYP2D6 substrate, and CYP3A4 inhibition profiles were retained negatives. This also suggested that these EO components can be used further or modified accordingly to more suitable derivatives in order to have more drug-like candidates.

The chemical structures of the chosen compounds from the essential oils were drawn first using the ChemDraw Ultra 8.0 software for the purpose of investigating their theoretical pharmacokinetics, which includes absorption, distribution, metabolism, and excretion (ADME). The accompanying descriptions were converted into the SMILES format. To assess the drug-like and pharmacokinetic characteristics of the selected compounds, we utilized the ADME tool provided by the SwissADME online server (http://www.swissadme.ch/, accessed on 1 June 2023), following a predefined procedure. To evaluate their toxicity profile, we employed the ProTox-II webserver (http://tox.charite.de/protox_II, accessed on 1 June 2023). This server utilizes various parameters, such as organ toxicity (hepatotoxicity), oral toxicity, and toxicological endpoints (cytotoxicity, mutagenicity, carcinogenicity, and immunotoxicity), to make predictions. From our analyses of the EO components using this tool we noted down important ADMET properties (Table 3).

### 3.6. Statistical Analysis

Multivariate analysis was performed according to the methodology described by [27], where the Minitab 17^®^ software (free version, Minitab Inc., State College, PA, USA) was used.

## 4. Conclusions

This paper investigated the chemical composition of the essential oils from six endemic specimens of *H. coronarium* in the Amazon region. The identification of variations in the chemical composition of *H. coronarium* essential oils contributes to the exploration of the species’ ecological and evolutionary aspects. By understanding how geographic and environmental factors shape the volatile profiles, we can gain insights into the adaptive mechanisms of *H. coronarium* and its interactions within its natural habitat. By uncovering the chemical diversity and complexity within this species, we open new avenues for the discovery of potential bioactive compounds and novel applications in various industries. The use of multivariate analysis enabled us to monitor the variability of the volatile compounds, both in terms of the compound classes and individual compounds, through the construction of a correlation matrix. This analytical approach allowed us to identify distinct chemotypes among the specimens studied, highlighting the intricate nature of *H. coronarium* volatile composition. The utilization of multivariate analysis techniques, along with the consideration of different plant organs, allowed us to unveil the complex variations and chemotypes present within this species. These results not only contribute to our knowledge of volatile compound profiles in the Amazon, but also have broader implications for ecological, pharmaceutical, and agricultural research. Overall, our findings advance the frontiers of knowledge in this field and lay the groundwork for future investigations into the chemical diversity and ecological significance of *H. coronarium*. In conclusion, our study contributes to a deeper understanding of the volatile compound profiles within the Amazon region and sheds light on the chemical diversity present in six endemic specimens of *H. coronarium*.

## Figures and Tables

**Table 3:** ADMET profile of the major compounds of different *H. coronarium* essential oils.

Constituents	1,8-Cineole	α-Pinene	β-Pinene	(E)-Caryophyllene
TPSA * (Å^2^)	9.23	0.00	0.00	0.00
Consensus log Po/w	2.67	3.44	3.44	4.24
Mol wt. (g/mol)	154.25	136.23	136.23	204.35
nRB	0	0	0	0
nOHA	1	0	0	0
nOND	0	0	0	0
WLOGP	2.74	3.00	3.00	4.73
Water solubility	Soluble	Soluble #	Soluble #	Soluble #
GI absorption **	High	Low	Low	Low
BBB permeant **	Yes	Yes	Yes	No
P-gp substrate **	No	No	No	No
CYP1A2 inhibitor **	No	No	No	No
CYP2C19 inhibitor **	No	No	No	Yes
CYP2C9 inhibitor **	No	Yes	Yes	Yes
CYP2D6 inhibitor **	No	No	No	No
CYP3A4 inhibitor	No	No	No	No
Log Kp (cm/s) (skin permeation)	−5.30	−3.95	−3.95	−4.44
Lipinski ***	Yes	Yes	Yes	Yes
Lipinski violation	0	1	1	1
Bioavailability score ***	0.55	0.55	0.55	0.55
Hepatotoxicity ****	No	No	No	No
Carcinogenicity ****	No	No	No	No
Cytotoxicity ****	No	No	No	No
Immunotoxicity ****	No	No	No	Yes
Mutagenicity ****	No	No	No	No
Predicted **** LD50 (mg/kg)	2480	3700	3700	5300
Toxicity class ****	V	V	V	V

ADMET: absorption, distribution, metabolism, excretion, and toxicity, lipophilicity *, pharmacokinetics **, drug likeliness ***, toxicological properties ****, TPSA: topological polar surface area, nRB: no. of rotatable bonds, nOHA: no. of H-bond acceptor, nOHD: no. of H-bond donor, WLOGP: water partition coefficient, GI absorption: gastrointestinal absorption, BBB: blood–brain barrier, P-gp: permeability glycoprotein, CYP: cytochrome P450, Toxicity class: (class I: fatal if swallowed (LD_50_ ≤ 5), class II: fatal if swallowed (5 < LD_50_ ≤ 50), class III: toxic if swallowed (50 < LD_50_ ≤ 300), class IV: harmful if swallowed (300 < LD_50_ ≤ 2000), class V: may be harmful if swallowed (2000 < LD_50_ ≤ 5000), class VI: non-toxic (LD_50_ > 5000)). # These components are non-soluble in water, as reported from real experimental data.

## Data Availability

Not applicable.

## Sample Availability

Samples of *Hedychium coronarium* J. Koenig. The essential oil in the Museu Paraense Emílio Goeldi is available from the authors.
